# Schistosomiasis and Intestinal Helminthiases in a Remote Area of Central Madagascar

**DOI:** 10.1155/jotm/3214987

**Published:** 2025-10-29

**Authors:** Gabriela Tapia-Veloz, Mónica Gozalbo, Elena Domingo, Danielle Aurore Doll Rakoto, Yeseul Kang, M. Teresa Galán-Puchades, Màrius V. Fuentes, María Trelis

**Affiliations:** ^1^Department of Pharmacy and Pharmaceutical Technology and Parasitology, Parasites & Health Research Group, Area of Parasitology, Universitat de València, Valencia, Spain; ^2^Department of Medicine and Public Health, Science of the Food, Toxicology and Legal Medicine, Universitat de València, Valencia, Spain; ^3^Doctoral School of Life and Environment Sciences, Faculty of Sciences, University of Antananarivo, Antananarivo 101, Madagascar; ^4^Department of Parasitology and Parasite Research Center, School of Medicine, Chungbuk National University, Cheongju, Republic of Korea; ^5^Joint Research Unit on Endocrinology, Nutrition and Clinical Dietetics, University of Valencia-Health Research Institute La Fe, Valencia, Spain

**Keywords:** intestinal helminths, neglected tropical diseases, poverty, schistosomiasis, soil-transmitted helminths, undernutrition

## Abstract

**Background:**

Schistosomiasis and intestinal helminthiases are significant public health issues that severely impact the development of children and adolescents in impoverished regions, such as the rural village of Soavinarivo in central Madagascar. These issues are often associated with deficient hygienic and sanitary conditions.

**Methods:**

A prospective cross-sectional study was conducted in October 2017 on children aged 3–15 years. A total of 114 stool samples were collected and analysed using microscopy and molecular techniques to identify schistosomiasis and intestinal helminthiases. Descriptive statistics assessed prevalence, and binary logistic regression identified risk factors for helminth infections.

**Results:**

Helminth prevalence was 88.6%, with *Schistosoma mansoni* being the most common (76.3%), followed by *Hymenolepis* (*= Rodentolepis*) *nana* (31.6%). Soil-transmitted helminths, including *Trichuris trichiura* and *Ascaris lumbricoides*, each affected 14% of participants. It should be noted that 5.3% of participants tested positive for *Taenia solium*. Undernutrition affected 66.7% of participants, with 58.8% suffering from stunting and 22.8% from wasting. Stunting was more likely in participants with *A. lumbricoides*. Risk factors for *S. mansoni* included being over 9 years old and working in rice fields. Not washing fruits and vegetables before eating and contact with pigs were related to *A. lumbricoides* infections. Clinical symptoms, such as abdominal distension and pain, were associated with *S. mansoni* infection.

**Conclusions:**

The prevalence rates of schistosomiasis and soil-transmitted helminthiases, highlighting *S. mansoni*, and its coexistence with undernutrition in the same population, are alarming. These findings underscore the urgent need to intensify current control strategies, which include deworming, provision of clean water, implementation of sewage systems and education on sanitation and hygiene.

## 1. Background

Schistosomiasis and intestinal helminthiases constitute a major public health challenge in structurally impoverished countries, especially in tropical and subtropical regions, in both rural and urban settings [1, 2]. Chronic or repeated infections during childhood are strongly associated with anaemia and stunting, which is the most common form of undernutrition in children under the age of five [1, 3]. Helminths mainly affect children, as the infective forms enter orally or penetrate through the skin, and at these ages, it is very common to walk barefoot and have close contact with each other, and with the ground or water when playing [1, 4]. Estimates of the prevalence of intestinal helminthiases among children in sub-Saharan Africa vary widely with a range of 9.1% to 90.3% [5].

In 2016, more than 29 million people in Madagascar needed humanitarian assistance for at least one neglected tropical disease (NTD). Schistosomiasis and soil-transmitted helminthiases (STH) were the main NTD affecting the Malagasy population [6]. The prevalence of STH on the island varies from 0% to 94%, being endemic in 111 of the 114 districts of the country [7]. According to the World Health Organization (WHO) protocol, only 69 districts with a prevalence of more than 20% were included in the mass drug administration (MDA) programs [8]. A study published by Hakami et al. in 2019 described the epidemiology of STH in 12 remote rural villages of Ranomafana National Park showing high prevalence rates: 74.7% for *Trichuris trichiura*, 71.4% for *Ascaris lumbricoides*, 33.1% for hookworms and 3.3% for *Strongyloides stercoralis*; it was estimated that 92.5% of people living in rural areas were affected by STH [9].

Schistosomiasis in Madagascar is widespread, and there is a substantial burden of this disease, making it a major public health problem. It has been reported in 106 of the 114 districts of the country, which are endemic for intestinal and/or urinary forms of the disease, with a prevalence reaching 89% [6]. A study in Marolambo district in 2021 found that of the 399 school-aged children examined, 93.7% were infected with *Schistosoma mansoni* [10].

Another intestinal helminthiasis widely distributed in the country is taeniasis caused by *Taenia solium,* a species with devastating effects on health causing human cysticercosis [11]. In NTD cartographic surveys conducted from October 2015 to February 2016 by the Ministry of Public Health of the country, 54 of the 114 districts were identified as coendemic for schistosomiasis and taeniasis, of which 7 districts were endemic only for *T. solium* [6].

In light of this background, the objective of this study is to assess the prevalence of schistosomiasis and intestinal helminthiases and the risk factors of infection among children and adolescents in the remote rural village of Soavinarivo, which is situated in the highlands of the centre of the island, an area marked by deficient hygienic-sanitary conditions, limited access to healthcare services, exclusion from national control programs and a lack of prior epidemiological data on these pathogens.

## 2. Methods

### 2.1. Ethics Statement

This study was approved by the Human Research Ethics Committee of the Universitat de València (Ref. H1506340075678) on October 5, 2017, and was conducted in accordance with the fundamental principles of the Declaration of Helsinki, the Council of Europe Convention on Human Rights and Biomedicine, and the UNESCO Declaration. Signed informed consent was obtained from all subjects involved in the study or their parents/legal guardians.

### 2.2. Study Design and Environment

A prospective cross-sectional observational study was conducted on children and adolescents aged 3–15 years, from the rural village of Soavinarivo, Madagascar. In October 2017, 114 stool samples were collected, one per participant, which were analysed by microscopy techniques and molecular methods for the identification, and characterisation in some cases, of schistosomiasis and intestinal helminthiases. Soavinarivo is located in the Ankazobe district of the Analamanga region; 122 km south of the capital, Antananarivo, in the central area of the island at 1249 m altitude [12]. In this region, a high mountain subtropical oceanic climate predominates, which is warm and temperate; the average annual temperature is 15.5°C [13]. To contextualise the reality of the village, it is worth mentioning that it has a difficult access due to its completely potholed dirt road. The population is young, rural, and most of the inhabitants are illiterate. Their main source of income is subsistence agriculture, with the vast majority dedicated to rice cultivation; combined or not with livestock. Some families own domestic animals such as pigs or chickens. The village has no electricity, water supply or sewage network. It is common to find rats and defecation usually takes place in the open.

### 2.3. Sample Collection

The inhabitants of Soavinarivo were contacted and invited to participate in collaboration with the local NGO for the Development of Children FAMI and the Spanish project Willka (Sekoly Willka, school for the village). All the children and adolescents who participated lived in the village and some of them attended the school. A general assembly was convened at the school for village families with children and/or adolescents. At this meeting, the actions to be carried out were detailed, and it was explained that participation in the study was voluntary. Families interested in participating had to fill out and sign an informed consent form.

Each legal representative received one kit per participant, which contained a plastic jar with an individually labelled lid, a spatula and a magazine sheet for sample collection. It was explained that the deposition had to be carried out on the magazine sheet to avoid contamination with the soil, and then, with the help of the spatula, three pieces of different parts of the deposition were taken, approximately 5 g of faeces, and placed in the container. The collected samples were immediately transferred to one of the school classrooms for processing and storage.

### 2.4. Epidemiological Survey

A personal interview was conducted with the legal guardians and a questionnaire was completed. Questions included (a) sociodemographic characteristics (age, sex, number of people residing in the household); (b) hygienic-sanitary habits (personal hygiene, environmental and food hygiene); and (c) clinical manifestations (diarrhoea, itching, headache, chills, fever, abdominal pain, bloating and flatulence).

### 2.5. Parasitological Assessment

Stool samples were processed in one of the school classrooms which was set up as an analysis laboratory. A single Kato–Katz thick smear per participant was performed, using fresh samples, for the detection, identification and quantification of schistosomes and intestinal helminths by light microscopy. The remaining faecal material was preserved in 70% ethanol and sent to the Parasitology Laboratory of the Faculty of Pharmacy and Food Sciences of the University of Valencia (Spain) for further analysis. Once in Spain, a stool sample of 5 g from each participant was concentrated and filtered for 5 min at 2500 rpm using Midi Parasep solvent-free (SF) devices (Apacor Ltd., Wokingham, UK). The sediment obtained from the centrifugation was divided into two aliquots, one that was used for DNA extraction, and the other that was fixed with 10% formalin in a 1:3 ratio for light microscopy examination of the concentrate.

Total stool DNA was extracted from 200 mg of concentrated samples, using the commercial QIAamp DNA Stool Mini Kit (Qiagen, Hilden, Germany) following the manufacturer's instructions and stored at −20°C until use. Molecular diagnosis was made using real-time multiplex PCR (qPCR) for the detection and identification of intestinal helminths using the Allplex GI-Helminth (I) Assay (GIPH) kit (Seegene Inc., Seoul, South Korea). This panel consists of a simultaneous qPCR for the detection of *Ancylostoma* spp.*, Ascaris* spp.*, Enterobius vermicularis, Hymenolepis* spp., *Necator americanus, Strongyloides* spp., *Taenia* spp. and *Trichuris trichiura.* Amplification was performed on the CFX96TM qPCR thermal cycler (Bio-Rad, Marnes-la-Coquette, France) using the CFX Manager IVD 1.6 software, in a 25-μL reaction volume containing 20 μL of the reaction mixture (5 μL of primers and probe (MOM), 5 μL of Anyplex PCR master mix (EM2), 8 μL of DNase/RNase-free water and 2 μL of DNA of internal control (IC)) and 5 μL of template DNA. Negative (DNase/RNase-free water) and positive controls were included in each assay. The results obtained were interpreted using the Seegene Viewer V3 software optimised for Allplex multiplex assays. Molecular detection of *Schistosoma* spp. was assessed by a qPCR protocol using the LightMix Modular *Schistosoma* spp. (TIB Molbiol, Berlin, Germany); the gene marker used by for the detection of the parasite is a specific 78 bp (base pair) fragment of the ITS-2 ribosomal RNA gene. The final qPCR reaction volume was 20 μL containing 15 μL of the reaction mixture (10.5 μL of ultrapure water, 0.5 μL of primers and probe and 4.0 μL of 1 × FastStart Essential DNA Probes Master Mix (Roche)) and 5 μL of template DNA. Positive and negative controls provided by the kit were included in each assay. The reactions were carried out in the CFX96TM thermal cycler (Bio-Rad, Marnes-la-Coquette, France), and the readings of the amplification curves were made using the CFX Manager IVD 1.6 software. Samples were considered positive if the cycling threshold (Ct) was below 37, as indicated by the manufacturer, considering Ct values between 20 and 35 to be optimal.

Characterisation of *Taenia* species detected by qPCR and/or microscopy was performed by partially sequencing the mitochondrial cytochrome C oxidase subunit 1 (*cox*1) gene using the primers described by Bowles and McManus [14]. PCR was performed in a total volume of 30 μL, containing 29 μL of the reaction mixture (19 μL of distilled water, 6 μL of 5 × HiPi Plus PCR Master Mix (EBT-1201, Elpis-biotech), 1.5 μL of each primer, 1 μL of Band Doctor 5X (SBD41-B10k, Solgent)) and 1 μL of template DNA, using the Super Cycler Gradient Cycler SC200, Kyratec.

The PCR products obtained after partial amplification of the *cox*1 gene were sequenced in both forward and reverse directions by the Sanger sequencing method by the Cosmo Genetech Company (Daejeon, Korea). The raw sequence files were assembled, and trimmed visually with Geneious Prime 2023.1.1.

### 2.6. Anthropometric Evaluation

Nutritional status was assessed with anthropometric measurement data (weight, height, age and sex) using the WHO Anthro (0–5 years) and WHO Anthro Plus (5–19 years) software.

### 2.7. Data Analysis

Binary logistic regression analyses were applied to evaluate the relationship between schistosomiasis and intestinal helminthiases and the independent variables: sex, age, nutritional status, sociodemographic factors, risk habits and the main symptoms reported by the surveyed population. The association between infection intensity, as quantified using the Kato–Katz technique, and both nutritional status and the most frequent symptoms was also analysed. Initially, a descriptive analysis of the sample was carried out using basic statistics such as percentages. Subsequently, a bivariate analysis was performed using chi-square tests for categorical variables, identifying preliminary significant associations. The magnitude of the association was expressed as odds ratios (OR) with a 95% confidence interval (CI). A *p* value < 0.05 was considered statistically significant. All variables were analysed using SPSS version 26.0 (Statistical Package for Social Sciences, Chicago, IL, USA) software.

## 3. Results

### 3.1. Sociodemographic Characteristics of the Study Participants

A total of 114 children and adolescents of the rural village of Soavinarivo participated in the study, 43.9% males and 56.1% females (Table 1). The mean age was 7 years, with a range from 2 to 15 years. The majority of the surveyed population did not attend school, and the households consisted of more than 4 members; 71.9% of the participants defecated and urinated in the open air, and only 33.3% used latrines. All houses in the village were surrounded by animal excrement, and 85.1% of the participants had contact with animals. The village lacks basic services such as electricity, a sewer system and drinking water, so that most of the inhabitants have to resort to using nonpotable water from rivers or lakes.

### 3.2. Occurrence of Intestinal Helminths

To optimise parasitological diagnosis, three analysis techniques were combined on faecal samples, light microscopy on the stool concentrates and on the thick smears of the Kato–Katz, and as a molecular technique a qPCR on the stool DNA; a multiplex panel for intestinal helminths and a single plex for schistosomiasis. Overall, 88.6% (101/114) of the participants were positive for helminths (Table 2). The most frequent helminths, after applying all diagnostic techniques, were the trematode *Schistosoma* spp., with a prevalence of 76.3% (87/114), followed by the cestode *Hymenolepis (= Rodentolepis) nana* (31.6%, 36/114). For nematodes, the highest percentage corresponded to the STH, *T. trichiura* and *A. lumbricoides*, with 14% (16/114) in both cases; the pinworm, *Enterobius vermicularis,* was detected in 10.5% (12/114) of cases. Finally, other intestinal helminths were also detected, but with lower infection rates: *Taenia* spp., *Necator americanus* and *Strongyloides* spp. It is worth noting, while panels sometimes provide a generic diagnosis, microscopy allows a complementary and specific identification that complements species assignment. The species *A. lumbricoides* was assigned to the positive samples of *Ascaris* spp. by qPCR as the most frequent in humans and synonymous with *A. suum* [15]. In the case of those positive for *Schistosoma* spp. by qPCR, Kato–Katz allowed the identification of *S. mansoni* by means of the characteristic morphology of the eggs observed.

With regards to co-infections, 34.2% (39/114) of the samples were infected with two helminth species, the most frequent combination being *S. mansoni* + *H. nana* (51.3%; 20/39). Infections involving three or more species were observed in 13.9% (18/114) of cases, most commonly the association *S. mansoni* + *H. nana* + *T. trichiura.* Only 13 (11.4%) participants tested negative in coproparasitological examinations (Table 2).

Comparing the results obtained according to the diagnostic technique employed (Table 3), it can be observed that qPCR detected more cases of *S. mansoni*, *T. trichiura* and *E. vermicularis* than the other techniques, and was also the only technique that detected *Strongyloides* spp. In the case of *Taenia* spp. and *N. americanus*, the results between techniques are concordant, and are very close in *A. lumbricoides* and *H. nana*. The Kato–Katz technique proved to be the least sensitive, although it allowed the intensity of the infection to be estimated. In *S. mansoni*, mild intensity was the most frequent (56.3%, 18/32), followed by moderate intensity (40.6%, 13/32) and only one case of severe intensity (3.1%, 1/32). All *A. lumbricoides* and *T. trichiura* infections were classified as mild intensity.

### 3.3. Molecular Characterisation of *Taenia solium*

A total of 6 samples tested positive for *Taenia* spp. by microscopy and qPCR. Generated CT values from qPCR amplification yielded a mean value of 32.7 (range: 30.5.0–36.9). Genotyping analyses were attempted for 5 of the 6 positive samples with the partial amplification of *cox*1 gene of the parasite and compared with the reference sequences (*T. solium* (NC004022), *T. saginata* (NC009938) and *T. asiatica* (NC004826)) reaching a 99.2%–100% identity rate with *T. solium.* All nucleotide sequences obtained in the present study were entered into the NCBI GenBank and made openly accessible with the following access numbers: PP259066, PP259067, PP259068, PP259069, PP259070.

### 3.4. Factors Associated With Intestinal Helminth Infections

Binary logistic regression analyses were conducted to assess the risk of schistosomiasis or intestinal helminthiasis based on the sociodemographic characteristics, the behavioural features and the nutritional status of the surveyed population. Age and gender were analysed as sociodemographic variables. Participants were divided into three age groups, pre-schoolers (≤ 5), schoolchildren (> 5–< 9) and preadolescents and adolescents (≥ 9). Infection with *S. mansoni* was significantly more prevalent in the preadolescent and adolescent group (90.5%, 38/42), who had a 4.75-fold higher likelihood of schistosomiasis (OR: 4.75; 95% CI: 1.37–16.44; *p*=0.016) compared to children younger than 5 years of age (Table S1). For the other intestinal helminth species, only an upward trend in prevalence was observed with increasing age. This pattern remained consistent across all species, except for *E. vermicularis* (13.9%, 5/42), which was more frequently observed in preschool and school-aged children (Table 4).

No significant association was identified between intestinal helminth infection and sex (Table S1). However, a higher prevalence of S*. mansoni* was observed in females (82.2% vs. 68%), and the same goes for the STH *T. trichiura* (17.2% vs. 10%) and *A. lumbricoides* (18.8% vs. 8%). In contrast, the intestinal helminths *H. nana* (28.1% vs. 36%) and *E. vermicularis* (7.8% vs. 14%) were more common in males (Table 4).

Regarding risk habits for schistosomiasis or intestinal helminthiasis (Table S1), the results revealed that children and adolescents who worked in rice fields were 7 times more likely to be infected with *S. mansoni* (95% CI: 2.55–19.24; *p* < 0.001) compared to those who did not. Children and adolescents who did not wash fruit and vegetables were 4.35-fold more likely to be parasitised by *A. lumbricoides* (95% CI: 1.31–14.46; *p*=0.010). In turn, participants who had contact with pigs were 13.36-fold more likely to be infected by this geohelminth (95% CI: 3.51–50.88; *p* < 0.001).

A total of 66.7% (76/114) of participants presented with undernutrition. Of these, 58.8% (67/114) were classified with stunting, and 22.8% (26/114) with wasting. Parasitisation with *S. mansoni* and STH was more frequent among individuals with stunting (Table 5). Analysis of schistosomiasis or intestinal helminth infection, stratified by parasitic species, against types of undernutrition revealed that participants infected with *A. lumbricoides* were 5.94 times more likely to present with stunting (95% CI: 1.28–27.56; *p*=0.007) compared to noninfected individuals (Table S1).

Finally, stratified binary logistic regression analyses were performed by parasitic species and the most frequent clinical manifestations (abdominal distension, abdominal pain and lack of appetite). The results revealed that children and adolescents infected with *S. mansoni* had a significantly higher likelihood of presenting with distension (OR = 4.06; 95% CI: 1.29–12.75; *p = 0.008*) and abdominal pain (OR = 3.68; 95% CI: 1.41–9.62; *p*=0.005) compared to those who were not parasitised (Table S2).

Analysis of infection intensity for *S. mansoni*, *A. lumbricoides* and *T. trichiura*, quantified using the Kato–Katz technique, showed no statistically significant associations with nutritional status or the presence of clinical symptoms.

## 4. Discussion

Schistosomiasis and intestinal helminthiases are a major health problem in children, historically affecting low- and middle-income countries, especially in tropical and subtropical endemic areas, such as Madagascar. This study focuses on vulnerable populations, such as children and adolescents in the village of Soavinarivo who live in remote areas with very limited resources. The strengths of the study include (i) the combined use of conventional (traditional microscopy) and molecular (qPCR) methods for the detection of schistosomiasis and intestinal helminthiasis; (ii) the genotyping of positive taeniasis samples to identify the species and (iii) the analysis of risk factors potentially associated with an increased likelihood of intestinal helminth infections.

The data obtained reveal a high prevalence of intestinal helminths (88.6%) in the children and adolescents surveyed. This finding is consistent with a previous study conducted in 12 rural villages near Ranomafana National Park in the Ifanadiana region, where 574 subjects participated and a prevalence of 92.5% was reported [9]. These results highlight the relevance of helminthiases as a serious public health problem in the Malagasy population, characterised by environmental and sanitary deficiencies, inadequate hygiene, overcrowding, low educational attainment, and poverty.

Schistosomiasis was the most common intestinal parasitic infection in the population studied. *Schistosoma mansoni* eggs were detected in faecal samples by light microscopy, 31.6% of cases in stool concentrates and 28.1% in thick smears of Kato–Katz. By qPCR, 71.9% of the participants obtained a positive result for *Schistosoma* spp. The high prevalence of *S. mansoni* observed in the village of Soavinarivo is comparable to another study carried out in the highlands of Central Madagascar in the Amoron'i Mania region, in which infection rates of 77.1% were found in schools of the villages of Ambositra and Ampasina [16]. Other studies conducted on the island reported a prevalence of schistosomiasis ranging from 35% to alarming figures reaching nearly 98% [10, 17, 18]. This high rate of infection found in the participants of the present study could be related to the fact that this population has the same habits, customs and ways of life, and they have never undergone any schistosomiasis control campaign. A similar situation was observed in a 2015 study in Western Madagascar, where a survey was conducted at randomly selected sites that had not received treatment for at least five years. The study revealed a prevalence of *S. haematobium* above 90% and an *S. mansoni* prevalence above 80% [19]. Our result also coincides with that published in a study that mapped the environmental suitability of schistosomiasis and the risk to human populations, finding a wide geographical distribution of the disease throughout the island. The Analamanga region, to which the village of Soavinarivo belongs, was identified as one of the areas with high environmental suitability and a considerable risk of transmission [20].

The second most prevalent helminth identified was the intestinal cestode *H. nana*, with a prevalence rate of 31.6%. Previous studies conducted in Madagascar have reported lower prevalence rates of this cestode, ranging from 0.4% to 9.1 [21, 22]. This discordance with our result may be due to several factors, such as differences in climatic conditions, environmental health and sanitation, prior control intervention, the socioeconomic status of the population and differences in host susceptibility to parasitic infections. In turn, it could be related to close contact with rodents, since they are the main reservoirs of *H. nana*. In addition, the choice of diagnostic techniques used in the different studies should be taken into account. Infections caused by *H. nana* are considered a threat to public health, as they are not only transmitted through contaminated food and water but also through person-to-person contact, which can lead to localised outbreaks. Additionally, this parasitic infection can cause autoinfection. Moreover, *H. nana* eggs may be present in water used for crop irrigation, potentially serving as an additional and common mode of transmission [22].

In relation to STH, the most frequent species were *T. trichiura* and *A. lumbricoides*. This finding is consistent with those obtained in the district of Marolambo, where 33.8% of school-age children had trichuriasis and 18.8% had ascariasis [23], and with another study carried out in the district of Moramanga, in which a prevalence of 16.1% for *A. lumbricoides* was found, although the prevalence of *T. trichiura* was lower, at 3.8% [24]. Research conducted in other areas of the island reported higher infection rates, ranging from 46% to 75% for one or both species [25, 26]. However, other studies reported prevalences of *A. lumbricoides* ranging from 0.4% to 4.4%, and *T. trichiura* ranging from 0.7% to 2.2%, which were lower than those observed in our study [19, 22]. These discrepancies in the prevalences can be attributed to several factors such as differences in soil types, moisture conditions, geography, environmental sanitation, as well as differences in altitude. Extreme environmental conditions influence the survival rates of STH eggs and larvae, as they are highly dependent on temperature: their development peaks between 28°C and 32°C and gradually declines until it ceases in extremely hot, cold or high-altitude environments [27–29]. Studies conducted in populations residing at high altitudes have shown low prevalences of STH, even in contexts of inadequate sanitation, poor hygiene and poverty [30, 31]. Soavinarivo is a village located in the highlands of the island at 1249 m above sea level and has an average annual ambient temperature of 15.5°C [12]. Therefore, climate and altitude could be contributing to the lower prevalence of STH.

In this study, traditional microscopy and qPCR techniques were used for better detection of schistosomiasis and intestinal helminthiasis. When comparing the results, qPCR was found to perform better, especially in the identification of *S. mansoni*. The discrepancy in detection rates between these techniques may be due to the low sensitivity associated with Kato–Katz due to variations in egg distribution among stool samples, which may have reduced detection rates in mild or recently acquired infections. In addition, performing only a Kato–Katz thick smear per individual is likely to have contributed to this difference in results, due to the small amount of stool analysed in the smear and since it is recommended to examine multiple Kato–Katz (in triplicate), prepared from the same stool sample or, better yet, from multiple stool samples to achieve optimal sensitivity [32]. Therefore, the application of molecular methods, whenever feasible, is particularly effective in identifying subclinical or low-intensity infections.

Genotyping results revealed that the taeniasis detected in the study population was caused by *T. solium*. Infection with this cestode occurs when a person eats raw or undercooked, infected pork, and therefore, it is a food-borne parasites of global importance. A study conducted by Lightowlers et al. in another region of the island, also identified only cases of *T. solium* [33]. The most serious complication associated with this species of cestode is neurocysticercosis, this pathology is common in Madagascar and leads to paediatric morbidity, causing more than 50% of epilepsy cases [34]. Conditions that facilitate the transmission of this tapeworm in humans are present in the country. These factors can include poor basic sanitation, poor hygiene practices such as open defecation, and the presence of free-roaming and free-feeding pigs, which increase the risk of ingesting *T. solium* eggs present in human faeces [11, 35]. In addition, the high endemicity of porcine cysticercosis found on the island, together with the general lack of slaughterhouses and insufficient inspection of pork, could be contributing to the Malagasy population contracting taeniasis [11, 34, 35].

Regarding risk factors, no statistically significant differences were found between schistosomiasis or intestinal helminth infections and sex; however, a higher prevalence of *T. trichiura*, *A. lumbricoides*, and, particularly, *S. mansoni* was observed in females. This result could be related to gender inequalities, as women perform most household chores, such as cooking, cleaning and laundry, which exposes them more frequently to fresh water. Importantly, the water sources they use in the village are not filtered or treated, and therefore may contain cercariae if they originate from untreated water. In addition, women frequently care for younger siblings and elderly relatives. According to data from the United Nations International Children's Emergency Fund (UNICEF), girls between the ages of 5 and 14 spend 40% more time on unpaid household chores, collecting water and firewood compared to boys their age [36]. On the other hand, participants older than 9 years were 4.75 times more likely to be infected with *S. mansoni* compared to those under 5 years of age. This finding is likely to be related to the fact that as age progresses so does exposure to parasites, as more time is spent outdoors and most of them are dedicated to the cultivation of rice fields. Additionally, throughout their lives, children and adolescents can accumulate parasitic infections due to repeated exposures, with reinfection being a prevalent phenomenon in these endemic regions. In many rural communities, older children are more involved in domestic and agricultural tasks, where they may come into contact with parasites. Working in rice fields was also associated with a higher likelihood of *S. mansoni* infection. This may be a consequence of the limited hygienic-sanitary conditions of the village, where 71.9% of the participants defecate and urinate in the open air, and only 38% use latrines, allowing the contamination of the fresh waters in which the aquatic snails vectors live. Schistosomiasis is more prevalent in areas with inadequate water supplies and poor sanitation and hygiene [37]. In a 2019 inspection in Madagascar, S*. mansoni* cercariae were found in both rice paddies and freshwater sources. Therefore, preteens and adolescents are likely to contribute to local transmission of *S. mansoni* through freshwater activities [2, 10].

In the case of *A. lumbricoides*, it was found that the consumption of unwashed fruits and vegetables increased the likelihood of infection. This could be because fruits and vegetables are contaminated with *A. lumbricoides* eggs using pig faeces as fertiliser or contaminated water [38]. Another significant risk factor for *A. lumbricoides* infection is contact with pigs. This finding may be related to the zoonotic potential of this nematode [39]. Several authors have provided evidence on cross-infection in both hosts by the genotypes of *A. suum* in humans and *A. lumbricoides* in pigs. In addition, a significant percentage of nematodes showing a hybrid pattern has been observed in both humans and swine [40, 41]. Findings of some studies support the proposal that *A. lumbricoides* and *A. suum* represent a single species [42]. However, another study mentions that *A. lumbricoides* and *A. suum* are genetically different, but are closely related and can hybridise. Therefore, *A. suum* also has the potential to infect humans [43, 44].

Despite international reports indicating an increase in overweight and obesity, even in low-income countries, certain regions of the world, such as sub-Saharan Africa, remain severely affected by undernutrition. Specifically, in Madagascar, the percentage of children under 5 years of age experiencing stunted growth is exceedingly high (> 30%) [45]. In the present study, high rates of undernutrition, including stunting and wasting, were documented. A study conducted among children in northern Madagascar reported a undernutrition prevalence of 41%, a figure lower than that observed in our study population, but still deeply concerning [46]. In 2022, data from three countries (Madagascar, Burkina Faso and Haiti) were analysed, revealing that Madagascar had the highest prevalence of undernutrition [47]. Previous research on the Malagasy population has documented alarmingly high rates of stunting, exceeding 69%, as well as wasting rates ranging from 11.2% to 18.8% [47–49]. In low-income countries, the main causes of undernutrition are economic hardship leading to poverty, which severely limits access to food, particularly animal products [50, 51], food insecurity exacerbated by climatic disasters (e.g. floods or droughts) and large family sizes. Another potential contributor to undernutrition, particularly stunting, is the presence of infectious diseases, as children and adolescents are particularly vulnerable due to the endemic circulation of various pathogens. In the present study, a significant association was shown between the presence of *A. lumbricoides* and stunting. Similar associations have been reported in previous studies [7, 52, 53]. This relationship could be explained by the negative impact on host feeding/nutrition causing diarrhoea, abdominal discomfort, loss of appetite and malabsorption of nutrients which are essential for the growth and cognitive development of children [7, 54, 55].

Regarding clinical symptoms, it was observed that children and adolescents infected with *S. mansoni* were more likely to present with abdominal distension and pain. These findings are consistent with previous literature, where long-term schistosomiasis has been associated with persistent inflammatory processes and the progression to intestinal and hepatosplenic alterations, which can manifest clinically as gastrointestinal symptoms [18, 56, 57]. In prior studies conducted in paediatric populations from endemic areas, abdominal pain has been identified as one of the most common symptoms, while distension, although less frequent, is typically associated with chronic infections and repeated exposure to the parasite over time [56, 58]. In this context, our results demonstrate that chronic schistosomiasis in childhood not only contributes to silent morbidity but also to clinically relevant gastrointestinal symptoms, underscoring the need to strengthen diagnostic and control strategies in these communities.

We are aware that the prevalence we report for *E. vermicularis* is underestimated, as the diagnostic techniques used are not recommended for this helminth. The optimal diagnostic method for *E. vermicularis* is the Graham test [59].

In Madagascar, the control of schistosomiasis and STH is carried out through public programmes and international collaborations, including the provision of praziquantel by the WHO and integrated MDA projects, alongside water, sanitation and hygiene (WASH) interventions. Nevertheless, significant challenges persist, such as limited treatment coverage—particularly in rural areas—the absence of formal educational programmes, and inadequate attention to vulnerable groups, factors that contribute to maintaining high infection rates. To strengthen control policies, it would be necessary to expand MDA coverage, with emphasis on hard-to-reach areas; more systematically integrate WASH programmes; include high-risk groups in prevention strategies; reinforce surveillance through predictive risk mapping and promote hygiene education. The coordinated implementation of these measures, in line with WHO guidelines and the Sustainable Development Goals, could significantly reduce the burden of schistosomiasis and STH, thereby improving child and community health in the most affected areas.

## 5. Conclusion

In conclusion, these results show the high parasitic pressure that this population has to endure in their day-to-day life and throughout their lives, due to hygienic-sanitary deficiencies and the social context, affecting child development, quality and life expectancy, as well as work productivity and the capacity for self-management in adults. Being an isolated rural area makes not only direct access to health care difficult, if not impossible, but also means the population cannot benefit from national helminthiases control programmes.

## Figures and Tables

**Table 1:** Sociodemographic characteristics of the studied population (*n* = 114).

Category	*N*	%
*Sex*		
Male	50	43.9
Female	64	56.1

*Age groups in years*		
≤ 5	36	31.6
> 5 < 9	36	31.6
≥ 9	42	36.8

*Schooled*		
Yes	33	28.9
No	57	50.0
NA	24	21.1

*Family size*		
< 4	9	7.9
≥ 4	105	92.1

*Open defecation or urination*		
Yes	82	71.9
No	32	28.1

*Use of latrines*		
Yes	38	33.3
No	76	66.7

*Animal excreta near the house*		
Yes	114	100
No	0	0

*Contact with animals*		
Yes	97	85.1
No	17	14.9

*Water for consumption*		
River or lake	103	90.4
Well	11	9.6

*Note: N*: number of cases; %: percentage of cases.

Abbreviation: NA, not applicable.

**Table 2:** Helminths detected in the surveyed population ranged by frequency.

	Positive cases		Negative cases	
	*n*	%	*n*	%
Intestinal helminths	101	88.6	13	11.4
*Schistosoma* spp.	87	76.3	27	23.7
*Hymenolepis (= Rodentolepis) nana*	36	31.6	78	68.4
*Trichuris trichiura*	16	14.0	98	86.0
*Ascaris lumbricoides*	16	14.0	98	86.0
*Enterobius vermicularis*	12	10.5	102	89.5
*Taenia* spp.	6	5.3	108	94.7
*Necator americanus*	5	4.4	109	95.6
*Strongyloides* spp.	1	0.9	113	99.1
Co-infections				
2 helminths	39	34.2		
≥ 3 helminths	18	15.8		

*Note: n*: number of cases; %: prevalence on the total samples (*n* = 114).

**Table 3:** Prevalence of helminths detected in the surveyed population according to the diagnostic technique.

Diagnostic technique	*Schistosoma mansoni*		*Ascaris lumbricoides*		*Trichuris trichiura*		*Necator americanus*		*Strongyloides* spp.		*Enterobius vermicularis*		*Hymenolepis nana*		*Taenia* spp.	
	*n*	%	*n*	%	*n*	%	*n*	%	*n*	%	*n*	%	*n*	%	*n*	%
Microscopy	36	31.6	12	10.5	5	4.4	3	2.6	0	0	3	2.6	33	28.9	6	5.3
Kato–Katz	32	28.1	8	7.0	5	4.4	0	0.0	0	0.0	0	0.0	11	9.6	2	1.8
qPCR	82	71.9	13	11.4	16	14.0	3	2.6	1	0.9	11	9.6	36	31.6	6	5.3

*Note: n*: positive cases; %: prevalence on the total samples (*n* = 114).

**Table 4:** Prevalence of more prevalent helminths by age group and sex.

	Age (years)						Sex			
	≤ 5 (*N* = 36)		> 5–< 9 (*N* = 36)		≥ 9 (*N* = 42)		Female (*N* = 64)		Male (*N* = 50)	
	*n*	%	*n*	%	*n*	%	*n*	%	*n*	%
Intestinal helminths	30	83.3	30	83.3	41	97.6	59	92.2	42	84
*Schistosoma mansoni*	24	66.7	25	69.4	38^∗^	90.5	53	82.8	34	68.0
*Hymenolepis nana*	9	25.0	10	27.8	17	40.5	18	28.1	18	36.0
*Trichuris trichiura*	4	11.1	4	11.1	8	19.5	11	17.2	5	10.0
*Ascaris lumbricoides*	5	13.9	5	13.9	6	14.3	12	18.8	4	8.0
*Enterobius vermicularis*	4	11.1	5	13.9	3	7.1	5	7.8	7	14.0

*Note: N*: samples tested; *n*: positive cases; %: prevalence per group.

^∗^Significant difference between groups (*p* < 0.05).

**Table 5:** Relationship between schistosomiasis and geohelminthiases and types of undernutrition.

	Stunting				Wasting			
	Yes		No		Yes		No	
	*n*	%	*n*	%	*n*	%	*n*	%
*Schistosoma mansoni*	51	58.6	36	41.4	19	21.8	68	78.2
*Ascaris lumbricoides*	14^∗^	87.5	2	12.5	1	6.3	15	93.8
*Trichuris trichiura*	12	75.0	4	25.0	4	25.0	12	75.0
*Necator americanus*	4	80.0	1	20.0	3	60.0	2	40.0
*Strongyloides* spp.	1	100	0	0	1	100	0	100

*Note: n*: number of cases; %: prevalence of association.

^∗^Significant association (*p* < 0.05).

## Data Availability

*T. solium* sequences obtained in the present study were entered into the NCBI GenBank and made openly accessible with the following access numbers: PP259066, PP259067, PP259068, PP259069, PP259070. The rest of the data presented in this study are available upon request from the corresponding author.

## Supporting Information

Supporting Information 1 Table S1. Binary logistic regression analyses for variables associated with the most common helminths identified.

Supporting Information 2 Table S2. Binary logistic regression analyses for symptoms associated with the most common helminths identified.
