# Supplementary material for: Schistosomiasis and Intestinal Helminthiases in a Remote Area of Central Madagascar
Source: J Trop Med. 2025 Oct 29;2025:3214987. doi: 10.1155/jotm/3214987 (PMC12588750; doi:10.1155/jotm/3214987)
Supplement: Supporting Information 1 — Table S1. Binary logistic regression analyses for variables associated with the most common helminths identified. [file 3214987.f1.docx]

**Table S1.** Binary logistic regression analyses for variables associated with the most common helminths identified.

|  | ***Schistosoma mansoni*** | | | | ***Ascaris lumbricoides*** | | | | ***Trichuris trichiura*** | | | | ***Enterobius vermicularis*** | | | | ***Hymenolepis nana*** | | | |
| --- | --- | --- | --- | --- | --- | --- | --- | --- | --- | --- | --- | --- | --- | --- | --- | --- | --- | --- | --- | --- |
| **Variable** | **No. *(n*)** | **OR** | **95% CI** | ***P*-value** | **No. *(n*)** | **OR** | **95% CI** | ***P*-value** | **No. *(n*)** | **OR** | **95% CI** | ***P*-value** | **No. *(n*)** | **OR** | **95% CI** | ***P*-value** | **No. *(n*)** | **OR** | **95% CI** | ***P*-value** |
| Age |  |  |  |  |  |  |  |  |  |  |  |  |  |  |  |  |  |  |  |  |
| <5 | 24 | – | – | – | 5 | – | – | – | 4 | – | – | – | 4 | 1.62 | 0.34–7.80 | 0.544 | 9 | – | – | – |
| >5<9 | 25 | 1.136 | 0.42–3.06 | 0.801 | 5 | 1.00 | 0.26–3.80 | 1.00 | 4 | 1.00 | 0.23 –4.35 | 1.00 | 5 | 2.09 | 0.47–9.46 | 0.336 | 10 | 1.16 | 0.40–3.32 | 0.788 |
| ≥9 | 38 | **4.75** | **1.37–16.44** | **0.016** | 6 | 1.03 | 0.29–3.72 | 0.960 | 8 | 1.88 | 0.52 –6.86 | 0.338 | 3 | – | – | – | 17 | 2.24 | 0.83-6.06 | 0.112 |
| Gender |  |  |  |  |  |  |  |  |  |  |  |  |  |  |  |  |  |  |  |  |
| Female | 53 | 2.27 | 0.94–5.47 | 0.068 | 12 | 2.65 | 0.80–8.80 | 0.111 | 11 | 1.86 | 0.60 –5.78 | 0.278 | 5 | – | – | – | 18 | – | – | – |
| Male | 34 | – | – | – | 4 | – | – | – | 5 | – | – | – | 7 | 1.92 | 0.57–6.46 | 0.292 | 18 | 1.63 | 0.72–3.73 | 0.239 |
| Work in rice field |  |  |  |  |  |  |  |  |  |  |  |  |  |  |  |  |  |  |  |  |
| Yes | 42 | **7.0** | **2.55–19.24** | **<0.001** | NA | – | – | – | NA | – | – | – | NA | – | – | – | NA | – | – | – |
| No | 45 | – | – | – | NA | – | – | – | NA | – | – | – | NA | – | – | – | NA | – | – | – |
| Water consumption |  |  |  |  |  |  |  |  |  |  |  |  |  |  |  |  |  |  |  |  |
| River or lake | 78 | 1.44 | 0.29–7.12 | 0.653 | 14 | 1.41 | 0.28–7.23 | 0.678 | 15 | 1.70 | 0.20–14.31 | 0.623 | 11 | 1.19 | 0.14–10.25 | 0.871 | 32 | 1.07 | 0.12–9.86 | 0.954 |
| Well | 9 | – | – | – | 2 | – | – | – | 1 | – | – | – | 1 | – | – | – | 4 | – | – | – |
| Handwashing before eating |  |  |  |  |  |  |  |  |  |  |  |  |  |  |  |  |  |  |  |  |
| Yes | NA | – | – | – | 4 | – | – | – | 5 | – | – | – | 7 | – | – | – | 12 | – | – | – |
| No | NA | – | – | – | 12 | 0.46 | 0.14–1.54 | 0.209 | 11 | 1.51 | 0.49–4.70 | 0.470 | 5 | 2.35 | 0.70–7.95 | 0.167 | 24 | 1.45 | 0.62–3.36 | 0.392 |
| Washing fresh produce |  |  |  |  |  |  |  |  |  |  |  |  |  |  |  |  |  |  |  |  |
| Yes | NA | – | – | – | 4 | – | – | – | 6 | – | – | – | 7 | NA | – | – | – | - | - | - |
| No | NA | – | – | – | 12 | **4.35** | **1.31–14.46** | **0.010** | 10 | 2.22 | 0.75–6.60 | 0.150 | 5 | NA | – | – | – | 1.13 | 0.30–4.24 | 0.861 |
| Contact with pigs |  |  |  |  |  |  |  |  |  |  |  |  |  |  |  |  |  |  |  |  |
| Yes | NA | – | – | – | 13 | **13.36** | **3.51–50.88** | **<0.001** | NA | – | – | – | NA | – | – | – | NA | – | – | – |
| No | NA | – | – | – | 3 | - | - | - | NA | – | – | – | NA | – | – | – | NA | – | – | – |
| Nutritional Status:  Stunting |  |  |  |  |  |  |  |  |  |  |  |  |  |  |  |  |  |  |  |  |
| Yes | 51 | 1.01 | 0.43–2.44 | 0.979 | 14 | **5.94** | **1.28–27.56** | **0.007** | 12 | 2.33 | 0.70–7.78 | 0.167 | NA | – | – | – | NA | – | – | – |
| No | 36 | – | – | – | 2 | – | – | – | 4 | – | – | – | NA | – | – | – | NA | – | – | – |
| Nutritional Status:  Wasting |  |  |  |  |  |  |  |  |  |  |  |  |  |  |  |  |  |  |  |  |
| Yes | 19 | 1.25 | 0.46–3.41 | 0.661 | 1 | 5.13 | 0.65–40.90 | 0.122 | 4 | 1.08 | 0.31–3.73 | 0.903 | NA | – | – | – | NA | – | – | – |
| No | 68 | – | – | – | 15 | – | – | – | 12 | – | – | – | NA | – | – | – | NA | – | – | – |

OR: Odds Ratio; 95% CI: Confidence Intervals; NA: Not Application. Statistically significant values are bolded.
