# Supplementary material for: Schistosomiasis and Intestinal Helminthiases in a Remote Area of Central Madagascar
Source: J Trop Med. 2025 Oct 29;2025:3214987. doi: 10.1155/jotm/3214987 (PMC12588750; doi:10.1155/jotm/3214987)
Supplement: Supporting Information 2 — Table S2. Binary logistic regression analyses for symptoms associated with the most common helminths identified. [file 3214987.f2.docx]

**Table S2.** Binary logistic regression analyses for symptoms associated with the most common helminths identified.

|  | ***Schistosoma mansoni*** | | | | ***Ascaris lumbricoides*** | | | | ***Trichuris trichiura*** | | | | ***Enterobius vermicularis*** | | | | ***Hymenolepis nana*** | | | |
| --- | --- | --- | --- | --- | --- | --- | --- | --- | --- | --- | --- | --- | --- | --- | --- | --- | --- | --- | --- | --- |
| **Symptoms** | **No. *(n*)** | **OR** | **95% CI** | ***P*-value** | **No. *(n*)** | **OR** | **95% CI** | ***P*-value** | **No. *(n*)** | **OR** | **95% CI** | ***P*-value** | **No. *(n*)** | **OR** | **95% CI** | ***P*-value** | **No. *(n*)** | **OR** | **95% CI** | ***P*-value** |
| Abdominal distension |  |  |  |  |  |  |  |  |  |  |  |  |  |  |  |  |  |  |  |  |
| Yes | 51 | 4.06 | 1.29–12.75 | **0.008** | 12 | 1.74 | 0.52–5.81 | 0.366 | 11 | 1.30 | 0.41–4.13 | 0.655 | 7 | 1.39 | 0.41–4.75 | 0.598 | 21 | 1.69 | 0.73–3.90 | 0.222 |
| No | 36 | – | – | – | 4 | – | – | – | 5 | – | – | – | 5 | – | – | – | 15 | – | – | – |
| Abdominal pain |  |  |  |  |  |  |  |  |  |  |  |  |  |  |  |  |  |  |  |  |
| Yes | 50 | 3.68 | 1.41–9.62 | **0.005** | 5 | 2.39 | 0.77–7.38 | 0.131 | 9 | 1.38 | 0.47–4.03 | 0.556 | 5 | 1.39 | 0.41–4.69 | 0.593 | 17 | 1.12 | 0.50–2.51 | 0.786 |
| No | 37 | – | – | – | 11 | – | – | – | 7 | – | – | – | 7 | – | – | – | 19 | – | – | – |
| Los of appetite |  |  |  |  |  |  |  |  |  |  |  |  |  |  |  |  |  |  |  |  |
| Yes | 10 | 1.62 | 0.33–7.91 | 0.549 | 4 | 3.75 | 0.98–14.36 | 0.069 | 3 | 2.38 | 0.56–10.14 | 0.241 | 1 | 1.41 | 0.16–12.27 | 0.753 | 1 | 6.42 | 0.78–52.64 | 0.083 |
| No | 77 | – | – | – | 12 | – | – | – | 13 | – | – | – | 11 | – | – | – | 35 | – | – | – |

OR: Odds Ratio; 95% CI: Confidence Intervals. Statistically significant values are bolded.
